# MiR-145-5p overexpression rejuvenates aged adipose stem cells and accelerates wound healing

**DOI:** 10.1242/bio.060117

**Published:** 2024-02-19

**Authors:** Chengcheng Li, Sen Ren, Hewei Xiong, Jing Chen, Tao Jiang, Jiahe Guo, Chengqi Yan, Zhenbing Chen, Xiaofan Yang, Xiang Xu

**Affiliations:** ^1^ Department of Hand Surgery, Union Hospital, Tongji Medical College, Huazhong University of Science and Technology, NO.1277 Jiefang Avenue, Wuhan 430022, China; ^2^ Department of Neurosurgery, Zhongnan Hospital of Wuhan University, Wuhan 430071, China; ^3^ Department of Emergency Surgery, Union Hospital, Tongji Medical College, Huazhong University of Science and Technology, Wuhan 430022, China; ^4^ Department of Dermatology, Wuhan No.1 Hospital, Wuhan 430000, Hubei, China

**Keywords:** Stem cells, MicroRNAs, Aging, BMPER

## Abstract

Adipose-derived stem cells (ADSCs) have been widely applied in translational and regenerative medicine. During aging, there is a recognized functional decline in ADSCs, which compromises their therapeutic effectiveness. Currently, the mechanisms of aging-induced stem cell dysfunction remain unclear, hence there is a need to elucidate these mechanisms and propose strategies for reversing this functional impairment. In this study, we found that ADSCs isolated from old donors (O-ADSCs) presented inferior phenotypes and decreased miR-145-5p levels compared to those from young donors (Y-ADSCs). To interrogate the role of miR-145-5p in ADSCs, gain- and loss-of-function assays were performed. The results indicated that miR-145-5p overexpression in O-ADSCs promoted cellular proliferation and migration, while reducing cell senescence. Further study demonstrated that miR-145-5p could regulate ADSCs function by targeting bone morphogenetic protein binding endothelial cell precursor-derived regulator (BMPER), which is a crucial modulator in angiogenesis. Moreover, *in vivo* experiments showed that miR-145-5p-overexpressing O-ADSCs accelerated wound healing by promoting wound re-epithelialization and angiogenesis. Collectively, this study indicates that miR-145-5p works as a positive regulator for optimizing O-ADSCs function, and may be a novel therapeutic target for restoring aging-associated impairments in stem cell function.

## Teaser

**Summary:** MiR-145-5p is decreased in adipose-derived stem cells (ADSCs) from the old donors (O-ADSCs), compared to that from young donors (Y-ADSCs). MiR-145-5p overexpression could promote O-ADSCs proliferation and migration, and further enhance their therapeutic effect for wound healing.

## INTRODUCTION

Mesenchymal stem cells (MSCs) have great potential for a range of clinical applications because they are immunocompatible, immunomodulatory, and can be differentiated into multiple cell lineages under specific culture conditions (Han et al., 2019; Krampera and Le Blanc, 2021). MSCs originate from the mesoderm and can be isolated from almost all tissues, including bone marrow, adipose tissue, and umbilical cord (Zhou et al., 2021). In particular, adipose tissues are plentiful and easily obtainable, making them desirable for harvesting. Furthermore, the abundant yield of isolating ADSCs and their efficient *in vitro* expansion capacity render them advantageous for both fundamental and applied investigations. In recent years, substantial progress has been achieved in ADSCs-based therapy (Bacakova et al., 2018). For example, ADSCs have osteogenic and adipogenic differentiation potential, allowing their application in bone tissue repair (Ho-Shui-Ling et al., 2018) and breast reconstruction (Fang et al., 2021), respectively. Additionally, ADSCs can also regulate the local tissue microenvironment by secreting various paracrine factors, such as soluble bioactive molecules and extracellular vesicles, which can accelerate wound healing and nerve regeneration (Chen et al., 2019a; Ren et al., 2019; Ren et al., 2022).

However, the function and subsequent therapeutic efficacy of stem cells can be severely affected by a vast range of adverse factors, including aging and disease in the donor. Previous studies demonstrated that ADSCs from type 2 diabetic patients and aged donors exhibit impaired proliferation capacity and mitochondrial dysfunction (Alicka et al., 2019; Sadie-Van Gijsen, 2021). There is also evidence that aging may attenuate the chondrogenic and osteogenic differentiation capacity of ADSCs, thus limiting their therapeutic effectiveness for degenerative bone and joint diseases (Marędziak et al., 2016). Furthermore, it has been observed that senescent stem cells exhibit an altered secretory profile, characterized by increased secretion of pro-inflammatory factors, referred as senescence-associated secretory phenotype (SASP). Therefore, it is necessary to uncover the underlying mechanisms of aging-related ADSCs dysfunction to identify novel targets for restoring the therapeutic efficacy of ADSCs.

MicroRNAs are short non-coding RNAs (19-25 nucleotides) that mainly regulate the expression of target mRNAs at post-transcriptional levels. Since the discovery of the first microRNA lin-4 in *Caenorhabditis elegans*, a vast number of studies have identified important biological roles for microRNAs, including in stem cell function and human diseases. (Saliminejad et al., 2019). There is also mounting evidence that microRNAs are involved in cellular senescence and age-associated diseases (Potter et al., 2021). For instance, the aging-related miR-155-5p and miR-195 are increased in MSCs from aged donors compared with MSCs from young donors. The abrogation of these two miRNAs in aged MSCs rejuvenates senescent MSCs and enhances cardiac function, by restoring balance in mitochondrial dynamics and reactivating telomerase activity in the cells, respectively (Hong et al., 2020; Okada et al., 2016). A recent high-throughput RNA sequencing (RNA-seq) study conducted by our team also identified many differentially expressed microRNAs between Y-ADSCs and O-ADSCs, highlighting the potential role of microRNAs as therapeutic targets (Ren et al., 2021).

The RNA-seq results identified miR-145-5p as a target that was decreased in O-ADSCs compared to Y-ADSCs, concomitant with an inferior functional phenotype. In this study, the RNA-seq results were verified by quantitative real-time polymerase chain reaction (qRT-PCR), reaching the consistent conclusion that miR-145-5p expression is lower in O-ADSCs than Y-ADSCs. However, whether and how miR-145-5p influences ADSCs function is still unclear. In this paper, we explored the effect of miR-145-5p on ADSCs function and its downstream mechanisms. The results may guide future therapeutic development to restore the function of O-ADSCs with potential clinical application.

## RESULTS

### Identification and characterization of ADSCs

**Fig. 1.**
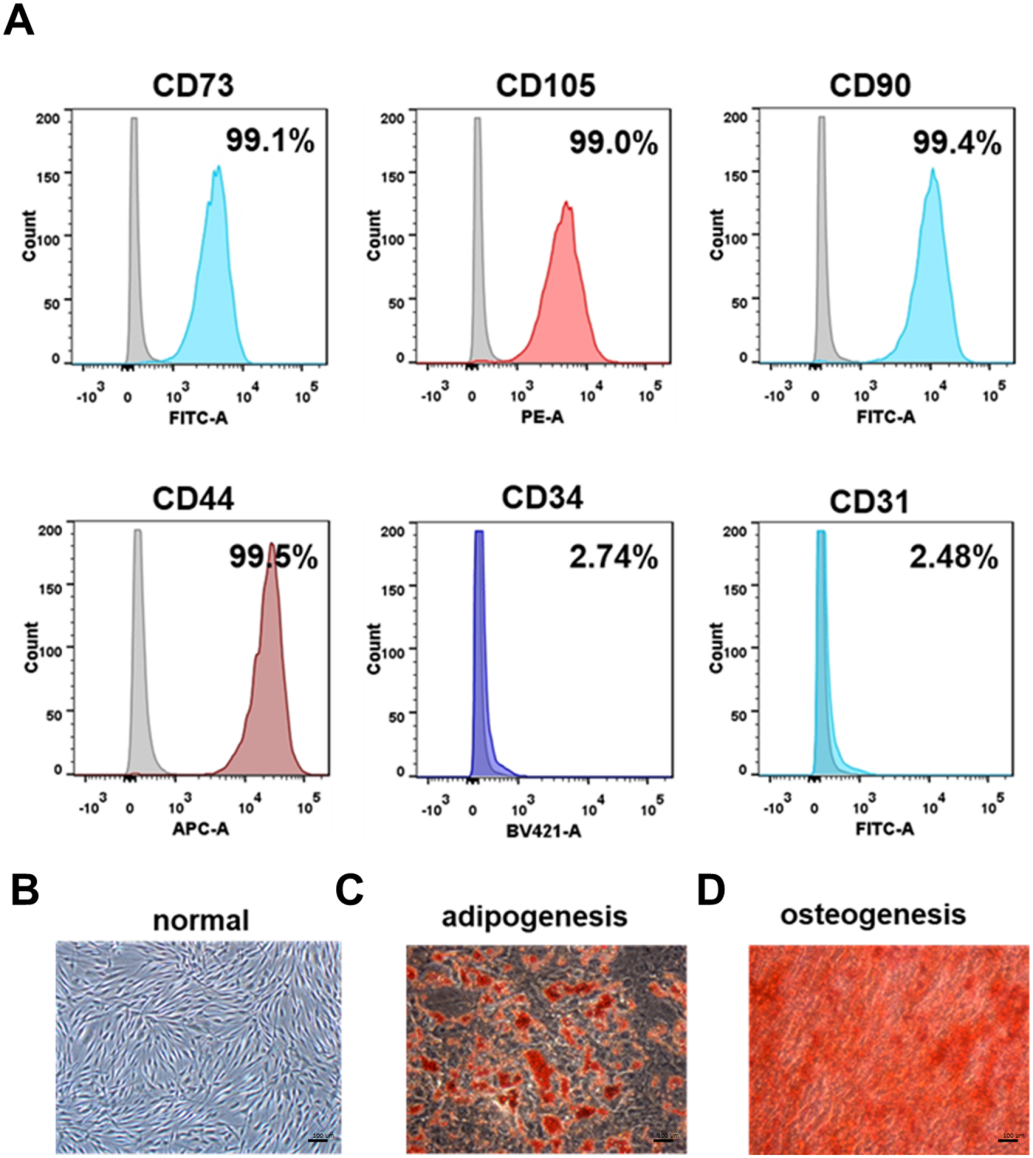
**Characterization of ADSCs isolated from human adipose tissue.** (A) Flow cytometric analysis of positive (CD73, CD105, CD90, CD44) and negative (CD34 and CD31) surface biomarkers for ADSCs. (B) Image of ADSCs under normal culture; scale bar: 100 μm. (C) Image of adipocytic differentiated ADSCs identified by Oil Red staining; scale bar: 100 μm. (D) Image of osteogenic differentiated ADSCs identified by Alizarin Red staining; scale bar: 100 μm.

Flow cytometric analysis and multilineage differentiation assay were performed to identify the isolated cells. Our flow cytometry analysis revealed that a significant proportion of isolated cells expressed ADSCs positive markers, including CD105 (99.0%), CD73 (99.1%), CD90 (99.4%) and CD44 (99.5%). Conversely, a minor fraction of isolated cells expressed ADSCs negative markers CD34 (2.74%) and CD31 (2.48%) (Fig. 1A). Moreover, we performed flow cytometric analysis for cells from donors of different ages, finding that MSC marker expression did not differ significantly between young and aged donors (Table S2). The undifferentiated cells were plastic-adherent and uniformly distributed in standard culture conditions, and exhibited a shuttle-shaped appearance in a whirlpool-like manner. (Fig. 1B). In contrast, after *in vitro* directional differentiation, lipid droplet formation (Fig. 1C) and calcium deposition (Fig. 1D) were observed in the cytoplasm of ADSCs, as evidenced by Oil Red O staining and Alizarin Red staining, respectively. Collectively, the characterizations of the isolated cells met the criteria for MSCs published by the International Society for Cellular Therapy (ISCT) (Dominici et al., 2006; Wang et al., 2020).

### O-ADSCs exhibit impaired proliferation and migration, as well as increased cellular senescence and DNA damage

The proliferation (Fig. S1A,B) and migration (Fig. S1E,F) of O-ADSCs were decreased 14% and 40%, respectively, compared with Y-ADSCs. Furthermore, the proportion of SA-β-gal-positive cells was ∼3-fold greater in O-ADSCs than Y-ADSCs (Fig. S1G,H). To evaluate DNA damage, we performed γ-H2AX staining, and found that the number of γ-H2AX-positive cells in the O-ADSC group was almost 1.6-fold more than that in the Y-ADSC group, indicating severe DNA damage in O-ADSCs (Fig. S1C, D). In summary, O-ADSCs demonstrate impaired proliferation and migration abilities, accompanied with a senescent phenotype and severe DNA damage.

### MiR-145-5p overexpression promotes O-ADSCs proliferation and migration, yet alleviated cell senescence

**Fig. 2.**
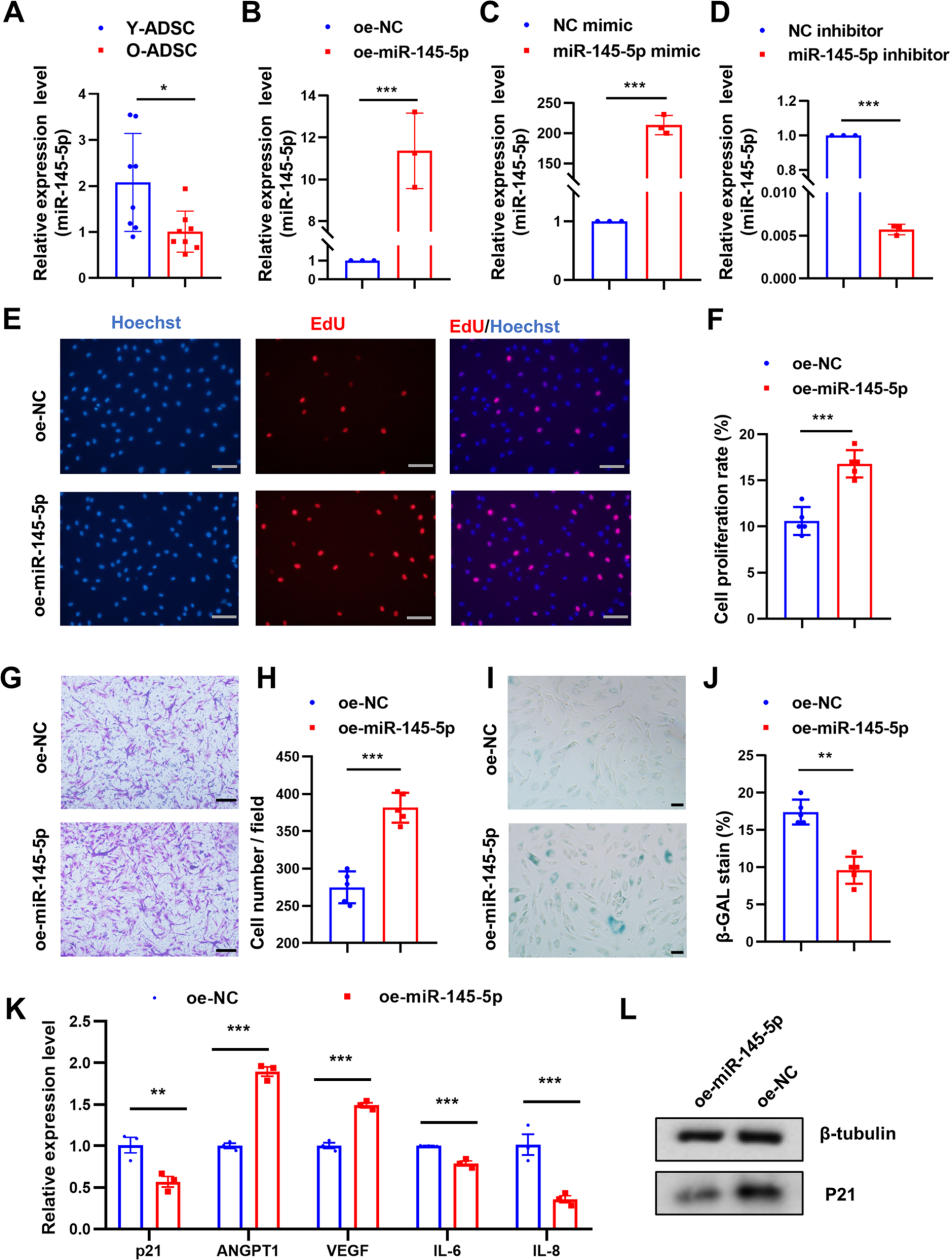
**miR-145-5p overexpression promotes O-ADSCs proliferation and migration, and rejuvenates cell senescence.** (A) Relative expression level of miR-145-5p in Y-ADSCs and O-ADSCs verified by qRT-PCR; *n*=8. (B) PCR analyses of the transfection efficiency of the miR-145-5p overexpression lentiviral vector; *n*=3. (C) PCR analyses of the transfection efficiency of the miR-145-5p mimic; *n*=3. (D) PCR analyses of the transfection efficiency of the miR-145-5p inhibitor; *n*=3. (E,F) Images and statistical analysis of EdU-positive cells in each group, the proliferative cells and cellular nuclei were stained with red and blue colors respectively; scale bars: 50 μm, *n*=5. (G,H) Images and statistical analysis of migrated cells in each group; scale bars: 100 μm, *n*=5. (I,J) Images and statistical analysis of SA-β-gal-positive (blue) cells in each group; *n*=5, scale bars: 50 μm. (K) PCR analysis of the expressional changes of RNAs in O-ADSCs after miR-145-5p overexpression. (L) Western blot analysis of the proteins levels in O-ADSCs after miR-145-5p overexpression. Data were presented as mean±s.d. unpaired Student's *t*-test was used. *P<0.05, **P<0.01, ***P<0.001.

To verify the differences in miR-145-5p expression in Y-ADSCs and O-ADSCs that were previously identified by RNA-seq (Ren et al., 2021; GSE174502), we performed qRT-PCR analysis. The qRT-PCR confirmed the RNA-seq results, demonstrating that miR-145-5p expression in O-ADSCs was significantly lower than in Y-ADSCs (Fig. 2A). To assess the role of miR-145-5p in O-ADSCs, we transfected a miR-145-5p-overexpressing lentiviral vector into O-ADSCs and validated the transfection efficiency by qRT-PCR (Fig. 2B). The proliferation rate in the oe-miR-145-5p group was almost 1.7-fold greater than that in the oe-NC group (Fig. 2E,F). Additionally, cell migration was almost 1.4-fold higher than in the oe-NC group (Fig. 2G,H). Moreover, the proportion of SA-β-gal-positive cells in the oe-miR-145-5p group was 8% less compared to the oe-NC group (Fig. 2I,J). Furthermore, miR-145-5p exerted inhibitory effects on the expression of inflammatory factor (IL-6 and IL-8) and senescence-associated marker P21, while simultaneously enhancing the expression of pro-angiogenic cytokines ANGPT1 and VEGF (Fig. 2K,L) In summary, miR-145-5p overexpression enhances the proliferation and migration of O-ADSCs, and partly rejuvenates their senescent phenotype.

### BMPER is a direct target of miR-145-5p

**Fig. 3.**
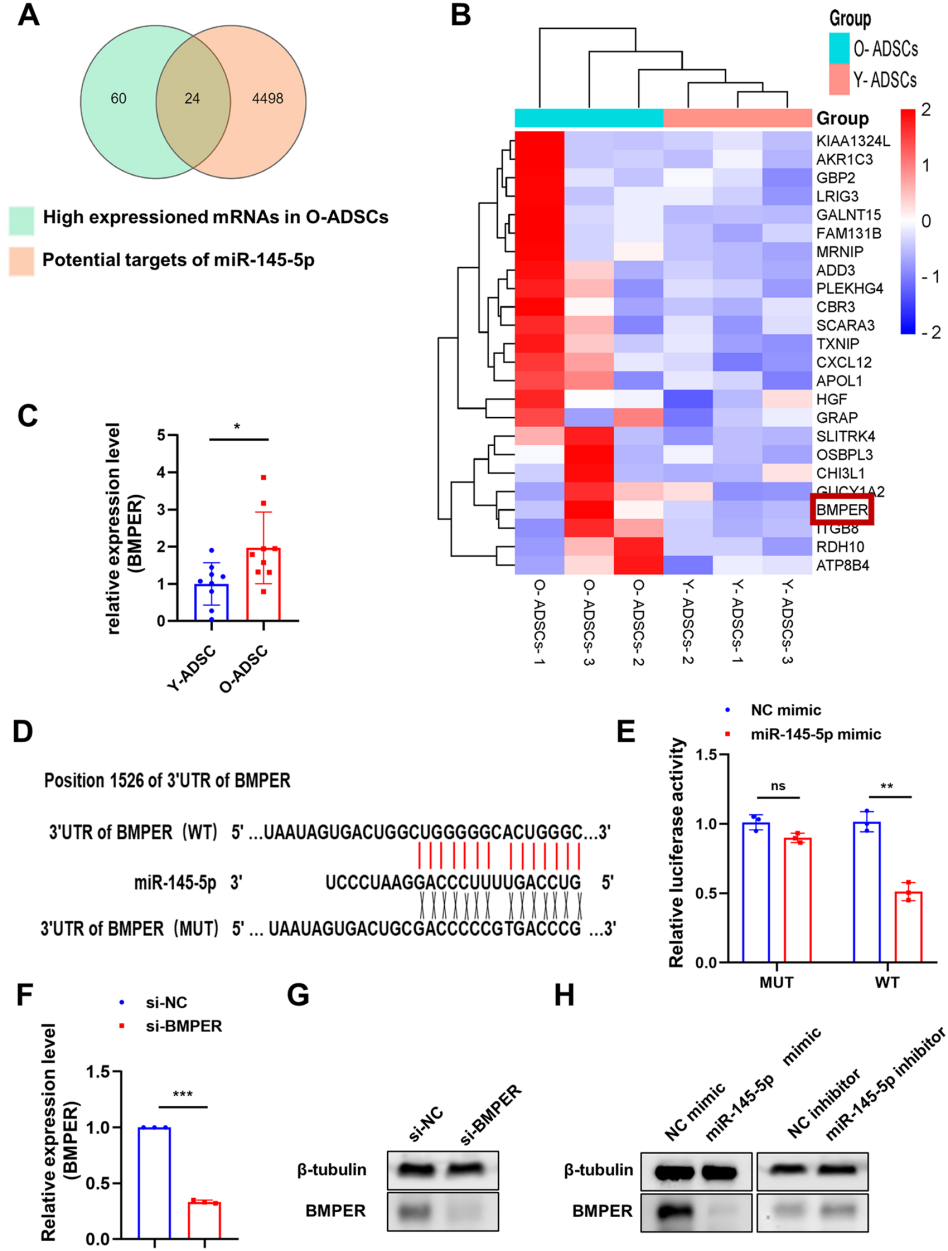
**BMPER is a direct target of miR-145-5p.** (A) Venn diagram describing the screened potential targets of miR-145-5p based on the RNA-Seq results and bioinformatic analyses. (B) Heatmap showing 24 overlapped genes. (C) PCR analyses of the expression level of BMPER in Y-ADSCs and O-ADSCs; *n*=9. (D) The sequences of BMPER-WT and BMPER-MUT were inserted into the psi-CHECK-2 vector and prediction of binding sequences between BMPER and miR-145-5p. (E) Luciferase activities of recombinant vectors with wildtype or mutated-type BMPER was examined in HEK-293 T cells by co-transfection with miR-145-5p mimic and mimic NC; *n*=3. (F) PCR analyses of the transfection efficiency of si-BMPER; *n*=3. (G) Western blotting analyses of BMPER expression in ADSCs after si-BMPER transfection; *n*=3. (H) Western blot analyses of BMPER expression in ADSCs after miR-145-5p mimic and miR-145-5p inhibitor transfection; *n*=3. Data were presented as mean±s.d.; unpaired Student's *t*-test was used in C and F. One-way ANOVA with Tukey's multiple comparisons test was used in E. ns, not significant; *P<0.05; **P<0.01; ***P<0.001.

To elucidate the downstream mechanisms by which miR-145-5p influences ADSCs function, we screened 84 mRNAs that were increased in O-ADSCs in comparison with Y-ADSCs. We also predicted 4522 potential targets of miR-145-5p using biological prediction websites (including RNAhybrid, TargetScan and miRanda; Fig. 3A). By highlighting targets that were identified by both approaches, 24 genes were clustered as candidate targets (Fig. 3B). Taking expression abundance and statistical significance into account, BMPER was identified as a target of interest for further study. We found that the expression level of BMPER in the O-ADSC group was ∼2-fold higher than that in the Y-ADSC group (Fig. 3C). Subsequently, luciferase reporters (pmirGLO-3′UTR of BMPER-WT and BMPER-MUT) were constructed (Fig. 3D) and transfected into the 293T cells. The luciferase activity of pmirGLO-BMPER-WT was significantly suppressed by miR-145-5p mimic, compared with NC mimic, while there were no significant effects on the luciferase activity of pmirGLO-BMPER-MUT in 293T cells (Fig. 3E). We also conducted western blot assays and found that BMPER expression in O-ADSCs transfected with miR-145-5p mimic was greatly decreased compared to those transfected with NC mimic. Conversely, BMPER levels in Y-ADSCs that were transfected with a miR-145-5p inhibitor were greatly increased compared to NC controls (Fig. 3H). In conclusion, these results indicate that miR-145-5p can inhibit BMPER expression by binding to the conservative complementary sequence of the BMPER mRNA 3′UTR.

### BMPER silencing rescues Y-ADSCs function following miR-145-5p inhibition

**Fig. 4.**
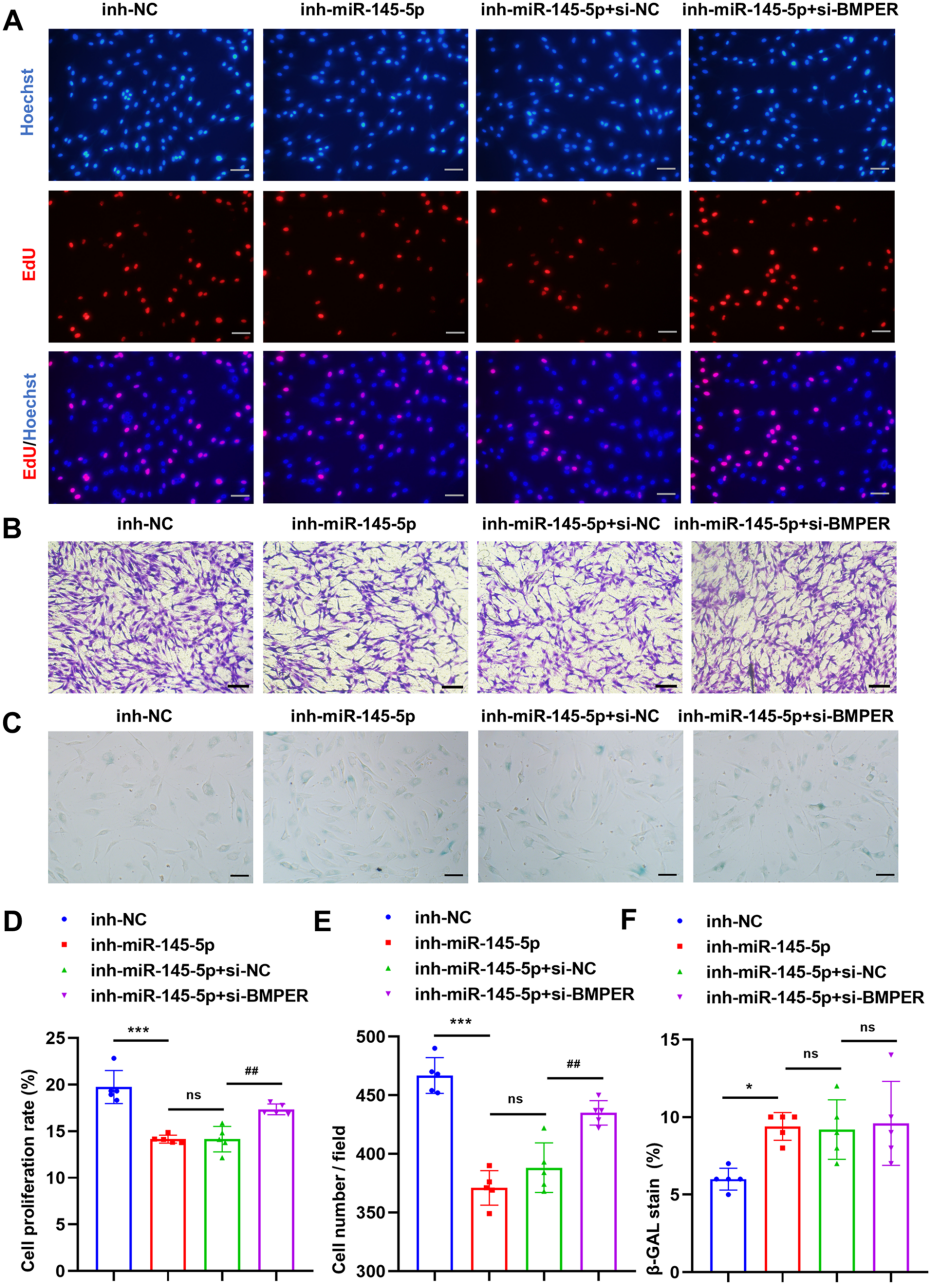
**BMPER silence in Y-ADSCs alleviates its function deterioration induced by the miR-145-5p inhibitor.** (A,D) Images and statistical analyses of EdU-positive cells (red) in each group; scale bars: 50 μm, *n*=5. (B,E) Images and statistical analyses of migrated cells in each group; scale bars: 100 μm, *n*=5. (C,F) Images and statistical analyses of SA-β-gal-positive (blue) cells in four groups; scale bars: 50 μm, *n*=5. Data were presented as mean±s.d. One-way ANOVA with Tukey's multiple comparisons test was used. ns, not significant; *P<0.05; **P<0.01; ***P<0.001; ^#^*P*<0.05; ^##^*P*<0.01; ^###^*P*<0.001.

**Fig. 5.**
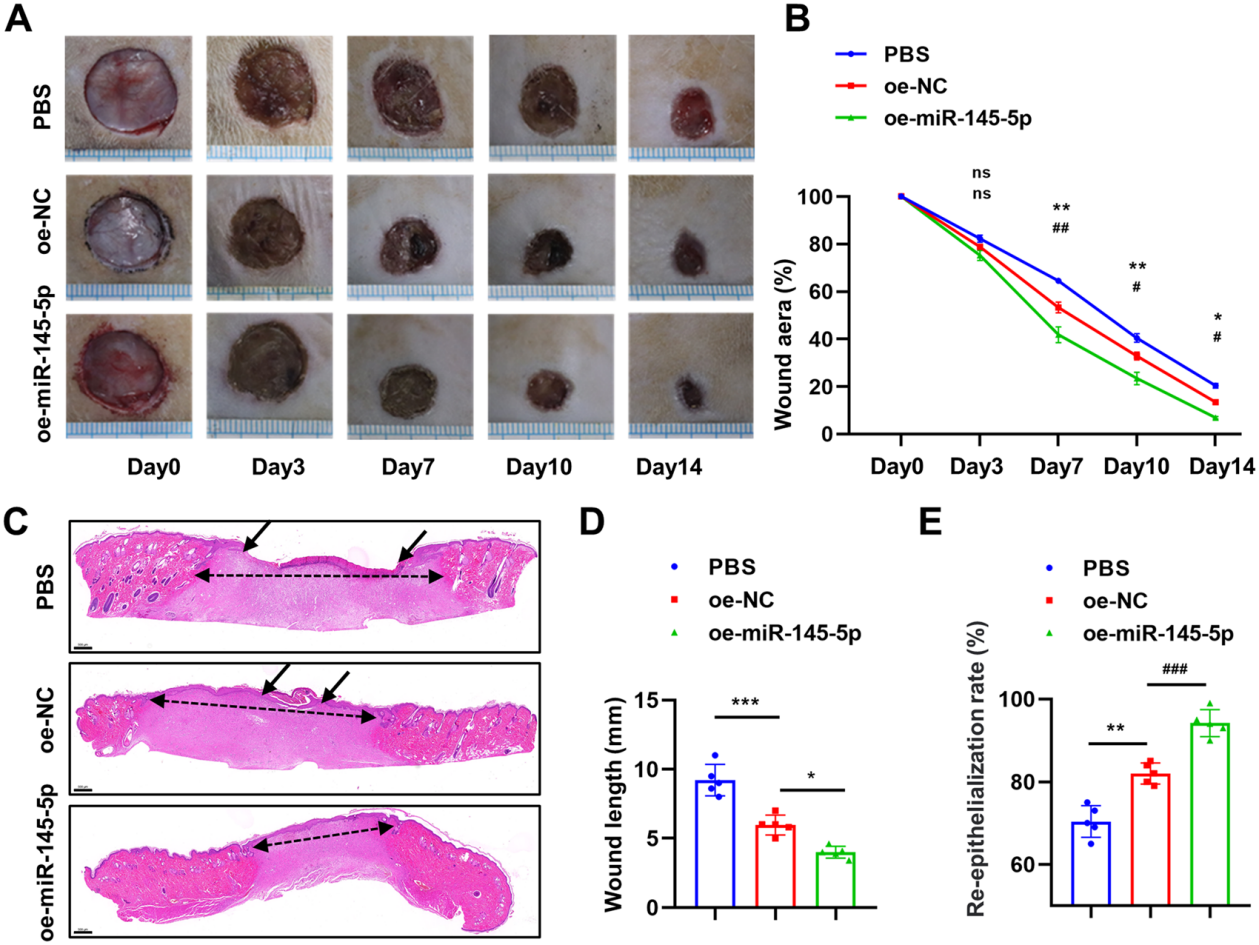
**miR-145-5p overexpression in O-ADSCs accelerates skin wound healing.** (A) The gross morphology of wound areas treated with PBS, oe-NC O-ADSCs (oe-NC) and oe-miR-145-5p O-ADSCs (oe-miR-145-5p) at different time points. (B) The statistical analyses of wound areas in each group at different time points. (C,D,E) The images of H&E staining and statistical analyses of wound length and re-epithelialization rates in each group at day 14, the single-headed arrows indicate the un-epithelialized areas, the double-headed arrows indicate the wound length. Scale bars: 500 μm. *n*=5, *P<0.05, **P<0.01, ***P<0.001, versus PBS. ^#^*P*<0.05, ^##^*P*<0.01, ^###^*P*<0.001, versus oe-NC, ns, no significance. Data were presented as mean±s.d. One-way ANOVA with Tukey's multiple comparisons test was used in D and E. Two-way ANOVA with Tukey's multiple comparisons test was used in B.

To determine whether miR-145-5p inhibition could impair Y-ADSCs function, we transfected miR-145-5p inhibitor (inh-miR-145-5p) or NC inhibitor (inh-NC) into Y-ADSCs. Y-ADSCs proliferation and migration were greatly impaired by miR-145-5p inhibition, decreasing by 6% and 20%, respectively (Fig. 4A,B,D,E). Furthermore, the proportion of SA-β-gal-positive cells in the inh-miR-145-5p group was almost 1.4-fold greater compared to the inh-NC group (Fig. 4C,F). Next, to determine whether BMPER silencing could rescue the functional defects induced by miR-145-5p inhibition in Y-ADSCs, we transfected si-BMPER or si-NC into Y-ADSCs that were simultaneously transfected with miR-145-5p inhibitor. The results showed that BMPER silencing partly reversed the functional deterioration of Y-ADSCs induced by miR-145-5p inhibitor, with ∼4% greater proliferation and ∼12% greater migration in the inh-miR-145-5p+si-BMPER group compared with the inh-miR-145-5p+si-NC group (Figs 4A, 5B,D,E). However, BMPER silencing did not reverse the increase in SA-β-gal-positive cells induced by miR-145-5p inhibitor in Y-ADSCs (Fig. 4C,F). In summary, our experiments demonstrate that miR-145-5p inhibition impairs Y-ADSCs function, with may be in part rescued by BMPER silencing.

### MiR-145-5p overexpression in O-ADSCs accelerates wound healing *in vivo*

A wound-healing animal model was used to assess the role of miR-145-5p in ADSCs *in vivo*. At 3-days post-wounding, there was no statistical difference in the wound area among the three groups (PBS, oe-miR-145-5p O-ADSCs, oe-NC O-ADSCs). However, at days 7, 10, and 14 post-wounding, the oe-miR-145-5p O-ADSCs group presented the smallest wound area, followed by the oe-NC O-ADSCs group, and the PBS group had the greatest wound area (Fig. 5A,B). Hematoxylin and Eosin (H&E) staining analysis showed that the oe-NC O-ADSCs group exhibited a greater re-epithelialization rate and shorter wound length than the PBS group, and the oe-miR-145-5p O-ADSCs group achieved the highest re-epithelialization rate and shortest wound length at the end of the experiment (Fig. 5C,D,E).

**Fig. 6.**
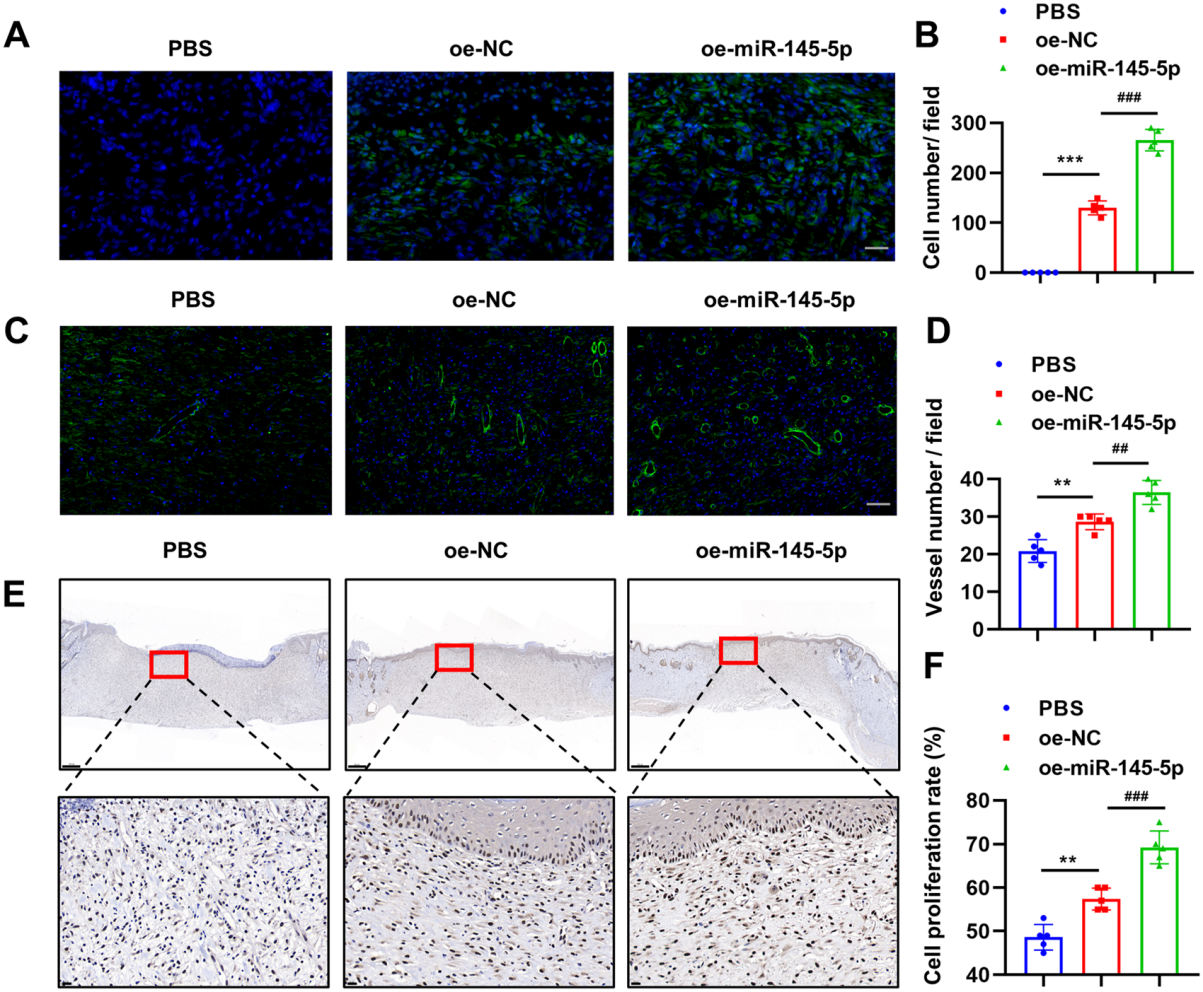
**miR-145-5p overexpression in O-ADSCs promotes cell proliferation and angiogenesis in wound beds.** (A,B) The images and statistical analyses of transplanted O-ADSCs in each group at day 14; scale bar: 50 μm. (C,D) The images and statistical analyses of α-SMA stained vessels in each group at day 14; scale bar: 100 μm. (E,F) The images and statistical analyses of PCNA stained cells in each group at day 14. Scale bars: 500 µm and 20 µm for low- and high-magnification images, respectively. *n*=5; *P<0.05; **P<0.01; ***P<0.001 versus PBS. ^#^*P*<0.05; ^##^*P*<0.01; ^###^*P*<0.001 versus oe-NC, ns, not significant. One-way ANOVA with Tukey's multiple comparisons test was used.

O-ADSCs also appeared to persist longer *in vivo* when miR-145-5p was overexpressed, as there were almost twice as many as cells remaining at day 14 in the oe-miR-145-5p O-ADSCs group compared with the oe-NC O-ADSCs group (Fig. 6A,B). In addition, angiogenesis at the dermal layer in the wound bed was investigated by α-SMA staining, and we observed the highest vascular density in the oe-miR-145-5p O-ADSCs group (1.8-fold that of the PBS group), followed by the oe-NC O-ADSCs group (1.4-fold that of the PBS group) (Fig. 6C,D). Moreover, the oe-miR-145-5p O-ADSCs group presented the highest cell proliferation rate (70%), followed by the oe-NC O-ADSCs group (57%), and the PBS group exhibited the lowest cell proliferation rate (49%) (Fig. 6E,F). Collectively, these results show that miR-145-5p overexpression significantly restores the therapeutic capabilities of O-ADSCs in would healing, by promoting re-epithelialization, neovascularization, and cellular proliferation in the wound bed.

## DISCUSSION

In the past, many preclinical and clinical studies have explored the development of ADSCs-based therapies, especially for wound repair (Hassanshahi et al., 2019; Jo et al., 2021). Mechanistically, ADSCs have the inherent ability to migrate to the wound site and differentiate into various skin cells, such as fibroblasts, endothelial cells and keratinocytes. In addition, ADSCs modulate the local microenvironment in the wound bed by secreting various bioactive molecules that act in a paracrine manner to promote an anti-inflammatory microenvironment, encourage progression from the inflammatory phase to the proliferative phase, and facilitate extracellular matrix remodeling (Mazini et al., 2020). Moreover, ADSCs-derived extracellular vesicles, encapsulating various beneficial biomolecules, can promote angiogenesis and collagen deposition, and thus accelerate the wound-healing process (An et al., 2021; Ren et al., 2019; Ren et al., 2022). However, the quality and function of ADSCs can be significantly impaired by aging, which reduces their therapeutic efficiency for wound healing (Birch and Gil, 2020; Boulestreau et al., 2020).

The cellular theory of aging postulates that human aging is a result of cellular aging in which more and more cells reach senescence, eventually resulting in the onset of age-associated diseases. Generally, senescence refers to a cellular response to endogenous and exogenous stresses, which triggers permanent cell cycle arrest and profound phenotypic changes (Birch and Gil, 2020; Mohamad et al., 2020). Several unfavorable factors interact synergistically to engineer cellular senescence, including DNA damage, mitochondrial dysfunction, and telomere attrition (Campisi and d'Adda di Fagagna, 2007). Aged stem cells are well known to have reduced capacity for repairing DNA damage (Pilzecker et al., 2019). In this study, γH2AX was used as a specific marker for DNA double-strand breaks to assess DNA damage in ADSCs. Consistent with previous studies, we observed elevated levels of γH2AX-positive cells in O-ADSCs, suggesting that O-ADSCs exhibit more severe DNA damage than Y-ADSCs. Furthermore, SA-β-gal, the most commonly used cellular marker of senescence, was more highly expressed in O-ADSCs than Y-ADSCs, suggesting a severe senescent phonotype. Given these important phenotypic differences between O-ADSCs and Y-ADSCs, there is a strong need to determine the mechanism of ADSCs senescence, to facilitate the development of strategies to rejuvenate senescent ADSCs.

Our previous research found that aging results in an altered transcriptome along with inferior function in ADSCs (Ren et al., 2021). There is also evidence that many microRNAs perform vital roles in aging and stem cell senescence (Choi et al., 2017; Dietrich et al., 2018; Potter et al., 2021). In this study, we focus on the role of miR-145-5p, which is decreased in O-ADSCs, in the function of ADSCs and their subsequent therapeutic effect in wound healing. As expected, miR-145-5p overexpression in O-ADSCs enhanced cell proliferation and migration, and partly reversed O-ADSCs senescence. By contrast, miR-145-5p inhibition in Y-ADSCs resulted in impaired function and a more senescent phenotype, suggesting that miR-145-5p may be involved in optimizing ADSCs function and protecting against senescence.

MicroRNAs can bind to the 3′-UTR of target mRNAs by interaction with their seed sequences, thus reducing target gene expression. Based on the mechanism mentioned above, we predicted and showed that BMPER is a direct target of miR-145-5p, and that BMPER silencing could partly rescue the functional deterioration of Y-ADSCs induced by miR-145-5p inhibition. BMPER is a biphasic extracellular secretory protein that regulates the BMP signal pathway activity and angiogenesis in a dose-dependent manner, with a low BMPER:BMP ratio promoting BMP signaling and angiogenesis, and vice versa (Kelley et al., 2009; Prosdocimo and Jain, 2011). Consistently, a previous study suggested that BMPER expression was restricted to endothelial cells and endothelial-derived cushions in the embryonic heart; endothelial migration was enhanced by a low BMPER concentration and inhibited by a high BMPER concentration (Dyer et al., 2014). Therefore, BMPER likely plays a crucial role in vascular development.

In our *in vivo* experiment, the wound area was smaller in the oe-NC O-ADSCs group than the PBS group, accompanied with higher vascular density and cellular proliferation. We ascribe this phenomenon to the paracrine effects of O-ADSCs. The higher vascular density at the wound bed in the oe-miR-145-5p O-ADSCs group is likely, at least in part, due to the downregulation of BMPER induced by miR-145-5p, which may promote angiogenesis at the dermal layer to provide oxygen and nutrients for the wound bed, thus accelerating the wound healing.

MiR-145-5p has been studied widely in a variety of contexts. For instance, in stem cell-based therapy, MSC-derived miR-145-5p-containing exosomes protected against chemotherapy-induced muscle loss (Cho et al., 2021) and inflammation in spinal cord-injury model (Jiang and Zhang, 2021). In cancer-related studies, miR-145-5p consistently functions as a tumor suppressor, inhibiting the proliferation, migration, and invasion of cancer cells (Chen et al., 2019b; Ding et al., 2020; Zhang et al., 2020). miR-145-5p also inhibited the proliferation and migration of vascular smooth muscle cells by targeting Smad4, which is a key signaling molecule of the TGF-β/Smad pathway (Li et al., 2018). In another cancer-related study, miR-145-5p inhibited tumor angiogenesis by targeting serpin family E member 1 (SERPINE1), a positive regulator for the VEGFR-2 signaling pathway (Teng et al., 2021). These studies seem to contrast our findings, which showed that the proliferation, migration and angiogenesis of ADSCs were increased with miR-145-5p overexpression. Given that there can be many targets of a specific microRNA, we speculate that microRNAs may play different roles in different cellular contexts by targeting different genes.

A recent study has revealed that miR-145-5p can mitigate high-glucose-induced cellular senescence by targeting p21, a cyclin-dependent kinase inhibitor, and subsequently activating the Erk/Akt signaling pathway (Su et al., 2023). In our study, both mRNA and protein levels of p21 decreased following miR-145-5p overexpression, accompanied by an increased cell proliferation rate, which is consistent with previous findings. Moreover, miR-145-5p inhibits inflammation-related factors TNF-α and IL-1β by suppressing the TLR4/NF-κB signaling pathway (Hui and Yin, 2018). In the present study, it was observed that miR-145-5p exhibited inhibitory effects on inflammation factors IL-6 and IL-8, while concurrently enhancing the expression of proangiogenic cytokines ANGPT1 and VEGF. Therefore, we hypothesize that miR-145-5p may attenuate the SASP and create favorable microenvironments for angiogenesis in wounds.

Previous studies have elucidated that many microRNAs regulate the wound-healing process, at different stages and by various mechanisms (Meng et al., 2018; Ross, 2021). For instance, microRNA-146a deficiency delayed wound healing in both normal and diabetic mice by enhancing inflammatory responses (Bi et al., 2022). For instance, microRNA-146a deficiency delayed wound healing in both normal and diabetic mice by enhancing inflammatory responses (Yan et al., 2022). In this study, we found that miR-145-5p overexpression in O-ADSCs restored aging-induced impairments in stem cell function and accelerated their wound-healing effects by promoting cell proliferation, re-epithelialization, and angiogenesis.

One of the major challenges of the modern world is the aging population and the accompanying increase in aging-associated diseases, which necessitates the development of effective and novel therapies that target aging. Due to their low immunogenicity, low tumorigenicity, multilineage differentiation, and immunomodulatory potential, ADSCs have emerged as a promising candidate for self-transplantation approaches to treating aging-associated chronic degenerative diseases. Currently, several factors pose challenges to the development of ADSCs-based therapies, including aged donors and consecutive cell passaging, which may expose ADSCs to senescence and hence limit their therapeutic effect and clinical application. In the present study, we propose miR-145-5p as a new target for rejuvenating O-ADSCs and improving their therapeutic efficacy for wound healing. Hence, the data presented in this study may contribute to the clinical translation of stem-cell-based therapies in the future.

In this study, we found that miR-145-5p is decreased in ADSCs from old donors compared to those from young donors. miR-145-5p overexpression promoted the proliferation and migration of old ADSCs, and enhanced their therapeutic effects for wound healing through targeting BMPER. Overall, our study proposes an aging-associated miR-145-5p/BMPER regulatory axis, providing a novel target for the improvement of stem-cell-based therapies using aged adipose stem cells.

## MATERIALS AND METHODS

### ADSCs isolation and culture

This study received approval from the Institutional Ethics Committee of Tongji Medical College, Huazhong University of Science and Technology, China (number 2022–S220), and adhered to the guidelines outlined in the Declaration of Helsinki for the use of patient samples. Informed consent was obtained from the parents of young donors and adult donors prior to their participation in the study. Adipose tissue samples were collected from patients who underwent skin-flap transplantation surgery and did not have any other systemic diseases. The ADSCs were cultured in Dulbecco's modified Eagle's medium (DMEM, OriCell, China) supplemented with 10% fetal bovine serum (OriCell, China), following a standardized protocol. Cells from passage 2 to 7 were used for the experiments. The demographic characteristics of the ADSCs donors included in this study are presented in Tables S1 and S2.

### ADSCs lineage identification

To conduct fluorescence-activated cell sorting (FACS) analysis, ADSCs obtained from various donors at passage 3 were subjected to an incubation process with fluorescein-conjugated mouse anti-human monoclonal antibodies. These antibodies specifically targeted cell surface markers such as CD31, CD34, CD44, CD73, CD90, and CD105 (all from Biolegend, San Diego, CA, USA). The incubation period lasted for 30 min at room temperature. Following this, the cells were washed with phosphate buffer solution (PBS) and subsequently resuspended in 200μL of PBS for FACS analysis. The Flowjo software was used to determine the percentage of cells exhibiting a positive signal.

In the multilineage differentiation assay, ADSCs obtained from various donors at passage 3 were chosen for the osteogenic and adipogenic differentiation assays, following the standard protocol provided by the manufacturer (Oricell, China). Adipogenesis capacity was assessed after 2 weeks of adipogenic culture using Oil Red O staining, while osteogenesis capacity was evaluated after 3 weeks of osteogenic culture using Alizarin Red staining.

### Lentiviral transfection

The lentivirus vector carrying green fluorescence protein (GFP) from Hanbio Biotechnology, China, was used to construct a stable overexpression system of miR-145-5p and a negative control. These vectors were then transfected into O-ADSCs for experimental purposes. In brief, O-ADSCs at passage 3 were seeded at a density of 1×10^5^ cells/well in a six-well culture plate. After overnight incubation, the virus stock solution was added to the ADSCs medium (Oricell, China) along with an appropriate concentration of polybrene and multiplicity of infection. After 12 h of transfection, the virus-containing medium was replaced with ADSCs medium. Once the cell confluence reached 70%, puromycin-containing ADSCs medium was used to select successfully transfected cells. These cells were then designated as oe-miR-145-5p O-ADSCs and oe-NC O-ADSCs, respectively.

### Mimic, inhibitor, and siRNA transfection

The miR-145-5p mimic and negative control mimic (NC mimic), miR-145-5p inhibitor (inh-miR-145-5p) and negative control inhibitor (inh-NC), si-BMPER and negative control si-RNA (si-NC) were synthesized by RiboBio, China. The specific sequences of these oligonucleotides can be found in Table S3. In brief, ADSCs at passage 3 were cultured in six-well plates at a density of 1×10^5^ cells/well. The oligonucleotides were transfected into the cells using the riboFECT CP Transfection Kit (RiboBio, China) following the manufacturer's instructions. The transfection efficiency was confirmed by qRT-PCR and western blot analysis. After 72 h of incubation, the ADSCs with different treatments were collected for further experiments.

### Real-time PCR analysis

In this study, both total RNA and microRNA were isolated using the Ultrapure RNA Kit (CWBIO, China). The extracted RNA samples were then reverse-transcribed into complementary DNA (cDNA) using the Prime-Script RT reagent Kit (TaKaRa, Japan). qRT-PCR was conducted following the manufacturer's instructions, using the SYBR Premix Ex Taq II (TaKaRa, Japan). The primer sequences used for qRT-PCR can be found in Table S4. The gene expression levels were determined using the 2−ΔΔCt method and were normalized to either GAPDH or U6.

### Western blotting

Total protein was extracted using RIPA buffer supplemented with a protease inhibitor cocktail, and the protein concentration was determined using the BCA protein assay (Aspen, China). An equal amount of total protein (20 μg) was separated by 10% SDS-PAGE and transferred onto PVDF membranes. The membranes were then blocked using a blocking buffer and incubated overnight with primary antibodies specific to BMPER (Santa Cruz, sc-377502, CA, USA) and β-tubulin (Proteintech, 10094-1-AP, China). Following this, the membranes were incubated with a secondary antibody labeled with HRP for 1 h. Subsequently, the membranes were incubated with the immobilon ECL substrate kit for 1 min and visualized using an imaging system.

### Cell proliferation and migration assay

In the cell proliferation assay, ADSCs from the same generations (between passages P4 and P6) were cultured in 96-well plates at a density of 7000 cells per well. Each experimental group consisted of five replicated wells. Following an overnight incubation, the cells were treated with the BeyoClick EdU-594 kit (Beyotime, China) for a duration of 3 h, following the manufacturer's instructions. Subsequently, the EdU-positive ADSCs (stained red) and all cells (stained blue) were visualized and quantified using a fluorescent microscope in the replicated wells of each group. The cell proliferation rate was determined by calculating the ratio of the number of red-stained cells to the number of blue-stained cells, multiplied by 100%.

In the cell migration assay, ADSCs from the same generations (between passages P4 and P6) were cultured in the upper chamber of a 24-well transwell insert (8.0μm pore size, Corning, AZ, USA). The seeding density was 3×10^4^ cells per well, using Dulbecco's modified Eagle medium without fetal bovine serum. The lower chamber was filled with 650μl of ADSCs medium. After 24 h of culture, the cells that migrated to the lower chamber were stained with crystal violet and visualized using a microscope. The number of migrated cells was then quantified for each group.

### β-galactosidase staining assay

ADSCs from the same generations (between passages P4 and P6) were cultured in 48-well plates at a density of 1.5×10^4^ cells per well. Each experimental group consisted of five replicated wells. Following an overnight incubation, the cells were subjected to staining using a Senescence β-Galactosidase Staining Kit (Beyotime, China). The presence of blue-stained cells was indicative of senescence-associated β-galactosidase (SA-β-gal) activity, which was observed under a light microscope. The proportions of SA-β-gal-positive cells were then determined for each experimental group.

### γ-H2AX staining assay

ADSCs were seeded in 48-well culture plates at 1×10^4^ cells/well, and each group had five replicated wells. Following an overnight incubation, the cells were stained using the DNA Damage Assay Kit (Beyotime, China), specifically the γ-H2AX Immunofluorescence method, as per the manufacturer's instructions. The proportion of γ-H2AX-positive cells (green) were calculated in each group.

### Dual-luciferase reporter gene assay

The binding sites between the miR-145-5p seed region and BMPER were analyzed using the RNAhybrid biological prediction website. Subsequently, the wild-type (WT) or mutant-type (MUT) sequence of the BMPER 3′-untranslated region (3′-UTR) were separately inserted into the pmirGLO luciferase reporter vector (Augct, China). The pmirGLO-BMPER-3′-UTR WT or pmirGLO-BMPER-3′-UTR-MUT constructs, along with either the miR-145-5p mimic or NC-mimic, were co-transfected into HEK-293T cells using Lipofectamine 2000 (Invitrogen, USA). Following a 36-h incubation period, the Renilla and firefly luciferase activities were measured using the Dual-Luciferase Reporter Assay System Kit (Promega Corporation, USA).

### *In vivo* wound-healing model

All animal experiments conducted in this study were granted approval by the Animal Care Committee of Tongji Medical College and were carried out in accordance with the ARRIVE guidelines. A total of fifteen healthy male Sprague Dawley rats, aged 10 weeks and weighing approximately 180-200 g, were obtained from the Experimental Animal Center at Tongji Medical College, Huazhong University of Science and Technology. The rats were randomly assigned to one of three groups: the PBS control group (PBS), the oe-NC O-ADSCs group (oe-NC), and the oe-miR-145-5p O-ADSCs group (oe-miR-145-5p), with each group consisting of five rats. Prior to the experiments, the rats were anesthetized using intraperitoneal injections of xylazine (0.25 mg/kg) and ketamine (0.025 mg/kg). Subsequently, a full-thickness cutaneous wound excision with a diameter of 16 mm was made on the back of each rat. Approximately 1.0×10^6^ oe-NC O-ADSCs or oe-miR-145-5p O-ADSCs were suspended in 100μl of PBS and subcutaneously injected at four points surrounding the wound, while the control group received an injection of 100μl of PBS only. Photographs of the wounds were taken on days 0, 3, 7, 10, and 14 post-wounding, and the wound area was measured in each photograph using Image J software. The wound area (%) was calculated by dividing the area of the actual wound by the area of the original wound and multiplying by 100%. Following the completion of sample collection, the rats were euthanized under anesthesia using the cervical dislocation method.

### Histological analysis

The wounds were collected on day 14 and subsequently underwent tissue fixation, embedding, and sectioning. The sections were then subjected to staining with H&E in order to analyze the wound length and re-epithelialization rate.

### Immunofluorescence analysis

In order to determine the localization and quantity of the remaining adipose-derived stem cells (ADSCs) that were injected into the wound on day 14, the sections were treated with a nuclear dye DAPI (Sigma-Aldrich, D9542-5MG, USA) and observed under a fluorescence microscope. The sections were then imaged, and the number of oe-NC O-ADSCs and oe-miR-145-5p O-ADSCs in each section was quantified using the Image J software.

To evaluate the vascular density of wound beds, the sections were incubated with α-SMA (Abcam, ab7817, USA) antibody overnight at 4°C, followed by incubated with fluorescein-conjugated second antibody (710,369, Invitrogen, USA) for 1 h at room temperature. The nuclei were then counterstained using the nuclear dye DAPI. Finally, the sections were visualized using a fluorescence microscope, and the number of blood vessels present in the dermal layer of each section was quantified using the Image J software.

### Immunohistochemistry analysis

To evaluate the cell proliferation of wound beds, the sections were incubated with Proliferating Cell Nuclear Antigen (PCNA) antibody (Abcam, ab7817, USA) overnight at 4°C. Subsequently, the sections were incubated with a second antibody conjugated with horseradish peroxidase (Beyotime, A0181, China) for 1 h at room temperature. Then the sections were imaged, and the proportion of PCNA-positive cells in each section was calculated using the Image J software.

### Statistical analysis

All data analysis were performed with GraphPad Prism software, version 8.0.2. Unpaired Student's *t*-test was applied to compare data between two groups, and one- and two-way analysis of variance (ANOVA) with Tukey's post-hoc test was applied to compare three or more groups. All data are presented as mean±standard deviation (s.d.) and statistical significance was set at *P*<0.05.

## Supplementary Material

10.1242/biolopen.060117_sup1 Supplementary information

Dataset 1.
