# Supplementary material for: MiR-145-5p overexpression rejuvenates aged adipose stem cells and accelerates wound healing
Source: Biol Open. 2024 Feb 19;13(2):bio060117. doi: 10.1242/bio.060117 (PMC10903265; doi:10.1242/bio.060117)
Supplement: Supplementary information [file biolopen-13-060117-s1.pdf]

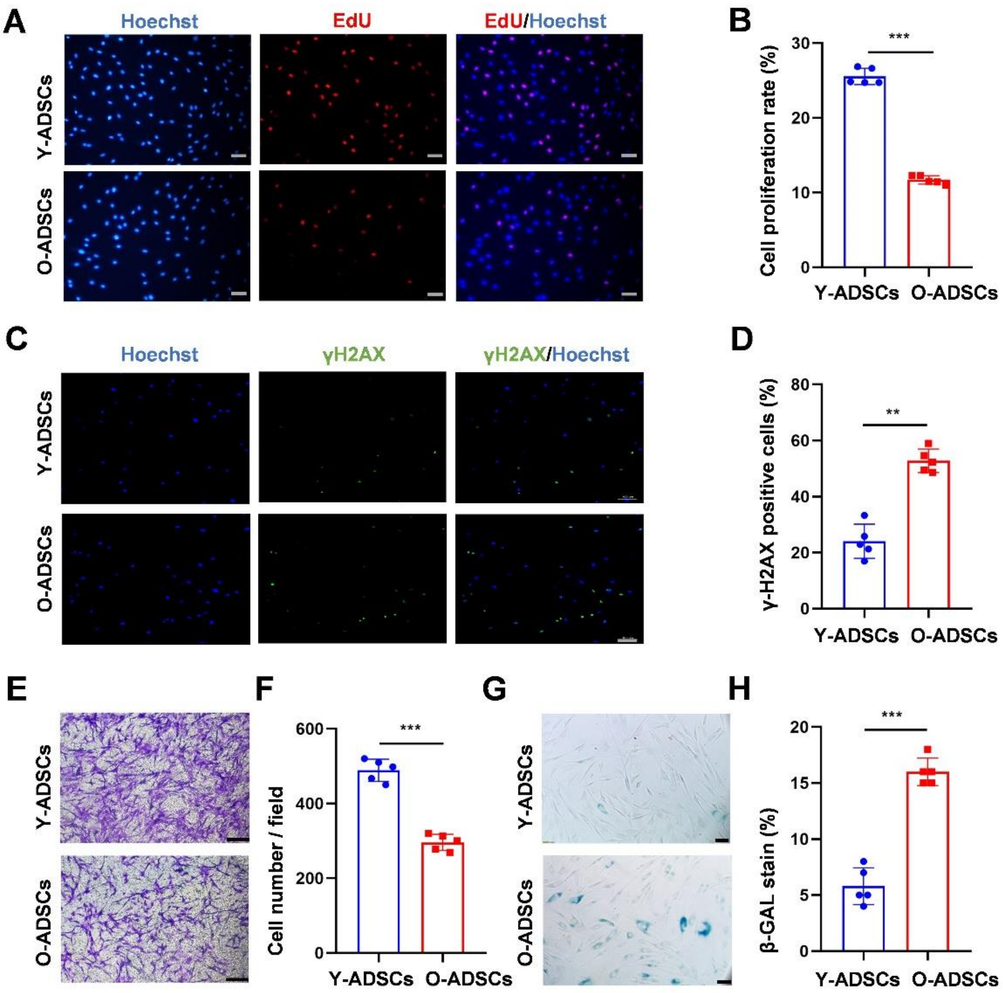

Fig. S1. O-ADSCs exhibit impaired proliferation and migration, yet excess cell senescence and DNA damage. (A, B) Images and statistical analyses of EdU-positive cells in each group; scale bar = 50um. (C, D) Images and statistical analyses of γH2AX-positive (green) cells in each group; scale bar = 100um. (E, F) Images and statistical analyses of migrated cells in each group; scale bar = 100um. (G, H) Images and statistical analyses of SA-β-gal positive (blue) cells in each group; scale bar = 50um. n=5, *p <0.05, **p <0.01, ***p <0.001. Data were presented as mean ± SD. Unpaired Student's t-test was used.

Biology Open • Supplementary information

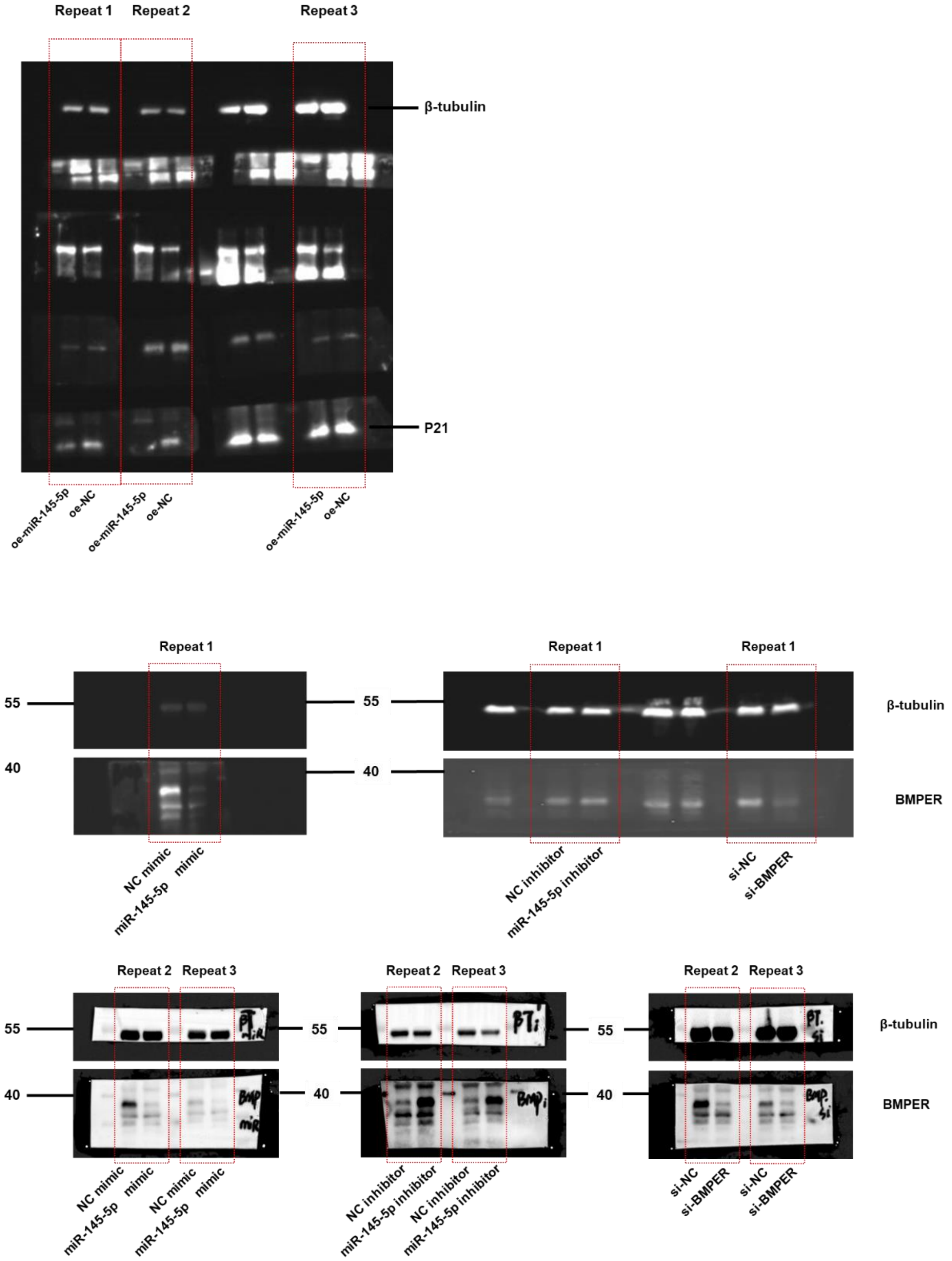

Fig. S2. Original blots

Biology Open • Supplementary information

Table S1. Basic characteristics of young donors and old donors used for RNA-seq.

	Young donors			Old donors		
Sample	Y-ADSC-1	Y-ADSC-2	Y-ADSC-3	O-ADSC-1	O-ADSC-2	O-ADSC-3
Age	7	10	11	52	51	56
BMI	23.67	21.51	23.81	22.84	25.61	24.49
SBP	110	112	NA	NA	130	115
DBP	70	84	NA	NA	70	76
FBG	5.31	5.17	5.21	4.9	5	4.61
TC	3.15	3.76	NA	3.15	3.33	NA
TG	1.43	1.08	NA	1.43	1.74	NA
BUN	4.15	4.16	3.68	NA	5.12	4.18
Cr	38.6	43.7	33	NA	72.9	62.4
HDL	NA	1.31	NA	1.17	0.83	NA
LDL	NA	1.89	NA	1.45	1.96	NA

Table S2. Basic characteristics of young donors and old donors used for qRT-PCR.

Variables	Young donors	Old donors	P value
Number	10	10	NA
Age	7.75±2.93	50.38±5.88	<0.001***
BMI	19.00±3.96	23.09±2.90	0.02*
SBP	115.00±12.61	125.45±10.60	0.08
DBP	75.00±7.98	82.18±9.51	0.12
FBG	4.87±0.39	5.27±0.76	0.21
TC	3.38±0.54	4.34±1.38	0.57
TG	0.88±0.29	1.50±0.53	0.22
BUN	4.01±1.05	4.26±0.63	0.52
Cr	37.321.56±4.17	59.68±9.31	<0.001***
HDL	1.36±0.06	1.05±0.21	0.19
LDL	1.63±0.37	2.47±1.24	0.63
CD31	0.87±0.89	1.80±1.56	0.42
CD34	1.29±0.88	2.32±1.65	0.39
CD44	99.27±0.35	99.50±0.2	0.37
CD73	99.40±0.26	99.57±0.32	0.53
CD90	98.83±1.17	96.80±4.33	0.48
CD105	99.37±0.12	99.23±0.25	0.45

Biology Open • Supplementary information

Table S3. The sequences of mimics, inhibitors and siRNAs.

Name	Direct	Sequence
MiR-145-5p mimic	5’-3’	GUCCAGUUUUCCCAGGAAUCCCU
	3’-5’	AGGGAUUCCUGGGAAAACUGGAC
NC mimic	5’-3’	UCACAACCUCCUAGAAAGAGUAGA
	3’-5’	UCUACUCUUUCUAGGAGGUUGUGA
MiR-145-5p inhibitor	5’-3	AGGGAUUCCUGGGAAAACUGGAC
NC inhibitor	5’-3’	UCUACUCUUUCUAGGAGGUUGUG
Si-BMPER	5’-3	GCGCUGUGUUGUUCAUUGUTT
	3’-5’	ACAAUGAACAACACAGCGCTT
Si-NC	5’-3’	UUCUCCGAACGUGUCACGUTT
	3’-5’	ACGUGACACGUUCGGAGAATT

Table S4. Primer sequences used for qRT-PCR.

Name	Sequence
MiR-145-5p	Forward 5’-GGATTCCTGGAAATACTGTTCT-3’
	Reverse 5’-TGGAACGCTTCACGAATTTGCG-3’
U6	Forward 5’-GGAACGATACAGAGAAGATTAGC-3’
	Reverse 5’-TGGAACGCTTCACGAATTTGCG-3’
BMPER	Forward 5’-GGGTGCGCTGTGTTGTTCATT-3’
	Reverse 5’-CTAAGGTGCTGGGGACAGGAG-3’
GAPDH	Forward 5’-AAAATCAAGTGGGGCGATGCT-3’
	Reverse 5’-TGGTTCACACCCATGACGAAC-3’

Dataset 1.

Available for download at
https://journals.biologists.com/bio/article-lookup/doi/10.1242/bio.060117#supplementary-data

Biology Open • Supplementary information
